# Prevalence of Colistin-Resistant, Carbapenem-Hydrolyzing Proteobacteria in Hospital Water Bodies and Out-Falls of West Bengal, India

**DOI:** 10.3390/ijerph17031007

**Published:** 2020-02-05

**Authors:** Taniya Bardhan, Madhurima Chakraborty, Bornali Bhattacharjee

**Affiliations:** National Institute of Biomedical Genomics, Kalyani 741251, West Bengal, India; tb1@nibmg.ac.in (T.B.); mc2@nibmg.ac.in (M.C.)

**Keywords:** hospital wastewater, colistin resistance, carbapenem-hydrolyzing, *bla*_NDM_ gene, West Bengal, India

## Abstract

Indiscriminate use of antibiotics has resulted in a catastrophic increase in the levels of antibiotic resistance in India. Hospitals treat critical bacterial infections and thus can serve as reservoirs of multidrug resistant (MDR) bacteria. Hence, this study was conducted to gauge the prevalence patterns of MDR bacteria in hospital wastewater. Water samples collected from 11 hospitals and 4 environmental sources belonging to 5 most-densely populated districts of West Bengal, India were grown on MacConkey and Eosin Methylene Blue agar. A total of 84 (hospital-associated = 70, environmental water sources = 14) isolates were characterized. The predominant species found in water from hospital-associated areas (HAA) were *Acinetobacter baumannii* (22.9%), *Escherichia coli* (28.6 %), and *Klebsiella pneumoniae* (25.7%). Greater than 75% of the HAA isolates were found to be *mcr-1* gene negative and colistinresistant. Meropenem non-susceptibility was also high among the HAA isolates at 58.6%, with the presence of the carbapenemase gene and *bla*_NDM_ in 67.1% of the non-susceptible isolates. Among the three predominant species, significantly higher numbers of *E. coli* isolates were found to be non-susceptible to meropenem ((80%), *p*-value = 0.00432) and amikacin (AK (90%), *p*-value = 0.00037). This study provides evidence for the presence of high numbers of colistin-resistant and carbapenem-hydrolyzing Proteobacteriain hospital wastewater.

## 1. Introduction

Antimicrobial resistance (AMR) is a global health challenge of the 21st century, and India contributes majorly to it [1,2]. India has a high burden of bacterial diseases, in addition to which, misguided overuse of antibiotics is also rampant [3]. It is one of the three leading consumers of antibiotics among low to middle-income countries across the world, with the highest usage of broad-spectrum penicillins [4]. However, medical practitioners still follow a pattern of usage in human infections, where carbapenems and polymyxins are considered to be last resort antibiotics in treatment. By contrast, in the animal production industry, polymyxins are routinely used as growth promoters [5,6]. Under such contrasting usage practices, both foodborne pathogens and high human usage of antibiotics can impact human health [7]. Hence, an understanding of the bacterial population distribution is imperative for better treatment design.

The use of antimicrobials is the highest in critical care units in hospitals that provide a selective environment for propagation of multidrug resistant (MDR) pathogens. These pathogens can effectively spread within hospital wards through infected medical devices and human contact and are also flushed out of hospitals through sewage [8]. Untreated or inadequately treated hospital wastewater has been reported to have the highest potential of dispersing such MDR pathogens to the community and the environment, where both the use of antimicrobials and antimicrobial pollutants maintain the selection pressure [9,10]. Once dispersed, these MDR pathogens can contribute to the spread of AMR against the commonly prescribed antimicrobials in a two-pronged manner; through multiplication and horizontal transfer of combinations of resistance-conferring genes to environmental isolates [11]. 

Reports from European countries and from the USA have highlighted the presence of higher proportions of colistin-resistance genes and extended-spectrum β-lactamase or carbapenemase-producing enteric bacteria in hospital effluents in comparison with urban wastewater [12,13]. Along similar lines, neighboring countries, such as Bangladesh and Singapore, have also reported prevalence of *bla*_NDM_ harboring bacteria in hospital wastewater and water collected from hospital-adjacent areas [14,15]. In the Indian context, reports are few and restricted to one or two hospitals [16,17,18]. So far, the spread of carbapenem-resistant Enterobacteriaceae and New Delhi Metallo-β lactamase-1 (NDM-1) in hospital wastewater out-falls has been explicitly probed only in New Delhi [19,20]. Colistin resistance is increasingly being reported by tertiary care hospitals in India; however, there are no reports on the prevalence rates in hospital wastewater [21].

A comprehensive understanding of the relative role of hospitals in environmental dissemination of AMR is essential for policy interventions to be put in place. Hence, this study was conducted in the fourth-most populous Indian state of West Bengal, and the objectives of this study were to understand: (i) the gram-negative species distribution, ii) antimicrobial resistance profiles, including colistin, and iii) *β-lactamase* gene carriage, and among the gram-negative bacterial isolates found in water samples collected from hospital wastewater out-falls and/or hospital ponds belonging to five districts, and contrast them with environmental water sources (EWS) in an unbiased manner, without selecting specific isolates resistant to any particular class of antimicrobial.

## 2. Materials and Methods

### 2.1. Collection of Samples and Isolation of Gram-Negative Bacteria

Water samples were collected from hospital-associated areas (HAA), which included untreated hospital wastewater from out-falls to community drains and natural water bodies within hospital premises belonging to 11 government-run hospitals from 5 districts of West Bengal with populations ranging from 4,496,694 to 10,009,781 inhabitants [22]. The districts were Howrah, Hoogly, Kolkata, Nadia, and North 24 Paraganas (Table 1). The names of the hospitals have been anonymized to maintain confidentiality. Institutional Ethical Committee Approval (RC/C/29032016) was taken to work on bacterial pathogens. The hospital water bodies were defined as those within the hospital premises with visible sewage pipelines traversing nearby. In parallel, samples from EWS, namely, four ponds/lakes from four of the same five districts, were also collected to serve as controls. These EWS were defined as natural water bodies without direct disposal of hospital effluents and in use by the community for household chores. 

Collection was carried out using the procedure described by Lamba et al. [19] with modification. Briefly, samples were collected in triplicate during the month of May 2018 using sterile 50 ml syringes. Particulate matter, if any were allowed to settle by sedimentation, and the pH were measured. After sedimentation, 10 µL of each sample was plated on Mueller Hinton agar (MHA (Himedia labs, Mumbai, India)) within 6–8 h of collection to be grown aerobically at 37 °C for 18 h in an incubator (Thermo Scientific, Waltham, MA, United States) to determine the total colony forming unit (CFU) counts. Simultaneously, specimens were also grown on both MacConkey with bile salts (MC) and Eosin Methylene Blue agar (EMB (Himedia labs) to determine the colony forming units (CFU) of gram-negative bacteria. 

Statistical power was calculated with sample size estimates of 6 isolates per hospital and 3 EWS isolates per district, and multiple testing correction was accounted for the significance level of 0.05. The expected differences in proportion of antibiotic non-susceptibility were 59%, which has been reported earlier [14], and a lower cutoff of 48%. This resulted in power values of 0.95 and 0.81, respectively, using the pwr.2p2n test package in R [23]. Accordingly, a total of 2–3 colonies of differing morphologies were isolated at random from both MC and EMB agar plates (Himedia labs) per sample. The colonies were subcultured in Mueller Hinton broth (Himedia labs) in a shaker incubator (Eppendorf, Hamburg, Germany), and 20% DMSO (Himedia labs) stocks were maintained at −80°C until further analyses.

### 2.2. Species Identification

DNA was isolated from subcultured isolates using the QIAamp DNA isolation kit (Qiagen, Hilden, Germany) and subjected to PCR amplification using the 16S- S-D-Bact-0008-c-S-20/ S-D-Bact-1391-a-A-17 primer pairs (Eurofins Scientific, Bengaluru, India) and Sanger sequencing. Amplicon sequences were queried against the National Center for Biotechnology Information (NCBI) *16S rRNA* gene database for species determination. 

### 2.3. Testing of Antimicrobial Susceptibility

The susceptibility profiles were generated using Kirby–Bauer disc diffusion assays, following Clinical & Laboratory Standards Institute (CLSI) guidelines (CLSI, 2017), and the antibiotics tested were aminoglycosides (amikacin (30 μg), gentamicin, (10 μg)), carbapenem (meropenem, (10 μg)), third generation cephalosporin (cefotaxime, (30 μg)), fluoroquinolone (ciprofloxacin, (5 μg)), penicillin+β-lactamase inhibitor (piperacillin/tazobactam, (100/10 μg)), and phenicol (chloramphenicol, (30 μg)) (Himedia labs).

### 2.4. Testing of Colistin Susceptibility

Colistin susceptibility was tested by the broth microdilution method using colistin sulphate (Himedia labs) and cation-adjusted Mueller Hinton II broth (CAMHB, Himedia labs) without supplementation of polysorbate-80 in polystyrene microtiter plates as per the CLSI- European Committee on Antimicrobial Susceptibility Testing (EUCAST) joint Polymyxin Breakpoints Working Group guidelines [24]. Three twofold dilutions ranging from 2 ug/mL to 8 ug/mL were used. *E. coli*, ATCC25922 and *Pseudomonas aeruginosa*, ATCC27853 were used as controls. Those isolates that exhibited growth at ≥4 µg/mL of colistin after 16–18 h, as detected by OD_600_, were considered to be resistant [25,26]. The assays were done in triplicate and were repeated three times to confirm the findings.

### 2.5. Genotyping of the β-Lactamase Genes bla_CTX-M_, bla_NDM_, bla_SHV_, bla_TEM_, and Plasmid-Borne Colistin Resistance Gene mcr-1

The four *β-lactamase* genes, namely, *bla*_CTX-M_, *bla*_NDM_*, bla*_SHV_, and *bla*_TEM_ and the *mcr-1* gene were amplified using PCR conditions and primer pairs that have been described before [27,28]. The positive controls used were laboratory isolate JNM10.C3 for the *β-lactamase* genes [27] and the *E. coli* NCTC13846 strain for the *mcr-1* gene amplifications. Sanger sequencing was carried out and the resulting sequences were queried against the Antimicrobial Resistance Database (ARDB) and the Bacterial Isolate Genome Sequence Database (BIGSdb).

### 2.6. Identification of Metallo-β-Lactamase (MBL) Producers by MBL Etest

All the isolates were tested for Metallo-β-Lactamase (MBL) by Etest (Himedia labs). The isolates were spread on MHA, and MBL Etest strips with meropenem(4–256 µg/mL) and meropenem inhibitior (meropenem-EDTA) (1–64 µg/mL) were applied and incubated for 16–20 h at 37 °C. A ratio of MRP to MRP-EDTA of >8 or a phenotype of no zone inhibition on the MRP coated side with inhibition zone on the MRP-EDTA side were considered to be MBL positive. This was in alignment with the manufacturer’s recommendations.

### 2.7. Statistical Analyses

Fisher’s exact test was carried out to identify statistically significant differences in species distribution, antimicrobial resistance phenotypes, and resistance genes among groups using the GraphPad Prism version 7.04 (GraphPad Software, La Jolla, CA, USA) [29]. A *p*-value of <0.05 was considered to be statistically significant. Multiple testing corrections were carried out independently for each hypothesis tested in this study, using the Benjamini–Hochberg method [30,31].

### 2.8. Availability of Data and Material

The *16S* V1-V8, *bla*_CTX_, *bla*_SHV_, *bla*_TEM_, and *bla*_NDM_ amplicon sequences have been submitted to GenBank, and the accession numbers are MK719774-MK719852, MN200677-MN200711, MN210360-MN210393, MN200712-MN200750, and MN200638-MN200750, respectively.

## 3. Results

### 3.1. Bacterial Load and Species Distribution

**Figure 1:**
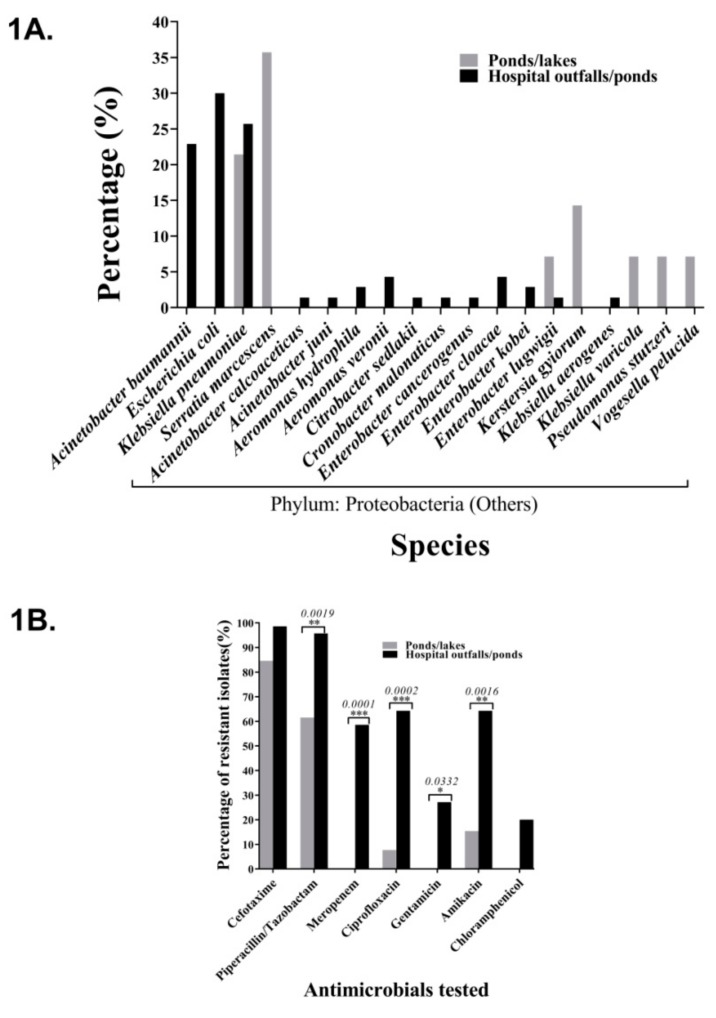
Differences in the distribution of bacteria between water samples collected from hospital-associated areas (HAA) and environmental water sources (EWS). (**A**) Species distribution percentages of isolates. (**B**) Antimicrobial resistance profiles for the antimicrobials tested. The asterisks indicate the statistically significant differences, and the *p*-values (Fisher’s exact test) are mentioned above.

The pH of the water samples varied between 7.44 to 8.56, with a median pH (±standard deviation) of 7.93 (±0.524) (Table 1). The total CFU/ml values (±standard deviation) varied between 1.0 × 10^3^ to 6.0 × 10^4^ (±2.2 × 10^4^) and there were no differences among groups. However, the hospital wastewater out-falls on an average had 10 times higher colony counts on MC (CFU/ml 1.4 × 10^4^ and 1.3 × 10^3^) and EMB agar (CFU/ml 2.4 × 10^4^ and 2.8 × 10^3^) compared to hospital ponds, while the EWS had 100 times lesser colony counts (MC: CFU/ml 1.4 × 10^4^ and 1.0 × 10^2^; EMB: CFU/ml 2.4 × 10^4^ and 2.0 × 10^2^). An average of 2–3 colonies from both EMB and MC agar were randomly selected from 10 hospital wastewater out-falls and 2 ponds, with the exception of W.KL.JN1 water samples, which when plated on MC agar had only 2 colonies. One isolate could not be identified and was removed from further analyses making the total count of isolates to be 70. From EWS, a total of 14 isolates could be obtained. All the isolates were found to belong to the phylum Proteobacteria, and Enterobactericeae was the predominant family (Genbank Accession numbers: MK719774–MK719852). Among the EWS isolates, *Serratia marcescens* (*n* = 5, 35.7%) was the dominant species, while *A. baumannii* (*n* = 16; 22.9%) and *E. coli* (*n* = 20; 28.6%) were found to be in the highest numbers among the HAA isolates. *K. pneumoniae* isolates were found both in HAA (*n* = 18; 25.7%) and in EWS (*n* = 3; 21.4%) (Figure 1A, Table S1). 

### 3.2. Antimicrobial Susceptibility Profiles

Out of the eight antimicrobials tested, cefotaxime had the highest non-susceptibility (intermediate or resistant) in both HAA (98.6%) and EWS isolates (84.6%). This was followed by piperacillin/tazobactam (95.7%) in the HAA isolates. Meropenem non-susceptibility was found in 58.6% of the HAA isolates and in none of the control isolates. One *Vogesella perlucida* isolate from the control group had to be excluded due to poor growth. The prevalence of resistance to piperacillin/tazobactam, meropenem, ciprofloxacin, gentamicin, and amikacin was significantly higher in hospital isolates (Figure 1B, Table 2). The three EWS *K. pneumoniae* isolates were found to be susceptible to meropenem, ciprofloxacin, gentamicin and chloramphenicol. However, all three were non-susceptible to cefotaxime, piperacillin/tazobactam, and colistin (Table S1). A total of 53 of the 70 (75.7%) HAA isolates were found to be resistant to colistin. Four out of five naturally resistant EWS *S. marcescens* isolates tested were also found to be resistant to colistin, further validating the results of the assay. Additionally, four meropenem-susceptible EWS *Klebsiella* spp. isolates and an *Enterobacter. ludwigii* isolate were also found to be resistant to colistin (Table S1). There were four HAA isolates resistant to all eight antimicrobials. 

**Figure 2:**
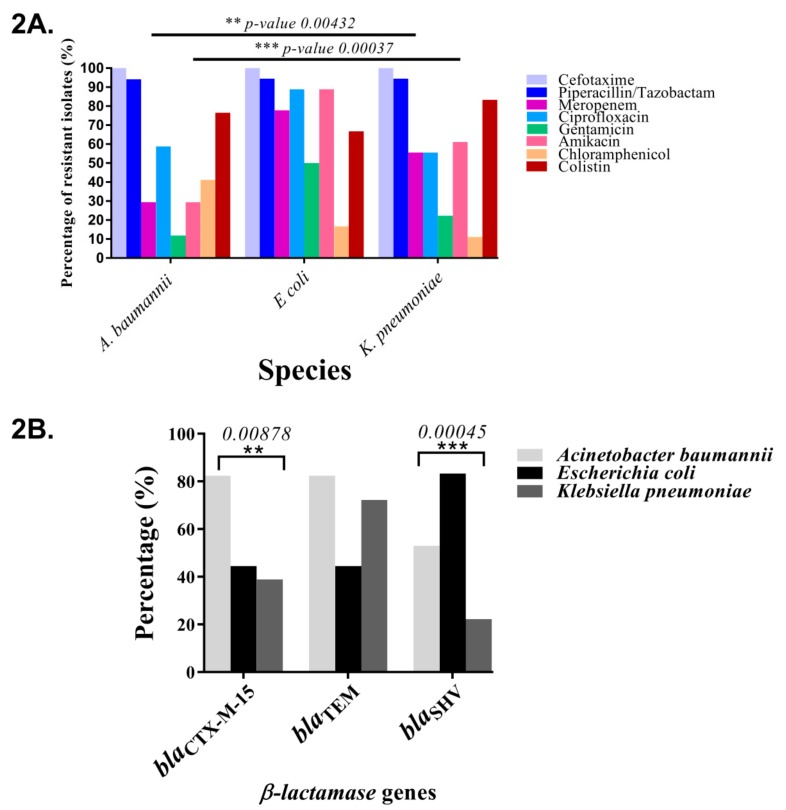
Differences in antimicrobial phenotype and β-lactamase gene carriage among the three dominant species found in HAA, namely, *A. baumannii*, *K. pneumoniae*, and *E. coli*. (**A**) Species-specific non-susceptibility profiles against the eight antimicrobials tested. (**B**) β-lactamase gene carriage differences. The asterisks indicate the statistically significant differences and the *p*-values (Fisher’s exact test) are mentioned above.

Among the HAA isolates, the species-specific distributions of susceptibility profiles were compared among *A. baumannii*, *E. coli,* and *K. pneumoniae* isolates because of high prevalence. All the *A. baumannii* and *K. pneumoniae* isolates were found to be non-susceptible to cefotaxime. Above 90%, all the isolates belonging to the three species were also non-susceptible to piperacillin / tazobactam. *E coli* isolates had the highest percentage of ciprofloxacin (90%), gentamicin (45%), amikacin (90%), and meropenem (80%) non-susceptibility, colistin resistance varied between 70% to 83.3% among the three species, and the *K. pneumoniae* isolates had the highest resistance (83.3%). However, susceptibility differences for only meropenem and amikacin were found to be statistically significant after multiple testing corrections (Figure 2A, Table 3, Table S1). Further, pairwise comparisons revealed that the frequency of both amikacin and meropenem non-susceptibility were lowest among the *A. baumanii* isolates and highest among the *E. coli* isolates, with the effect being more prominent in the case of amikacin (Table S2).

### 3.3. Carriage of Candidate β-Lactamase and mcr-1 Genes among the Pathogenic HAA A. baumannii, K. pneumoniae, and E. coli Isolates

The genes *bla*_CTX-M_, *bla*_SHV_*,* and *bla*_TEM_ were amplified and sequenced, as described previously [27]. Out of a total of 54 HAA *A. baumannii, E. coli*, *and K. pneumoniae* isolates, 55.6% (*n* = 30) were found to harbor the extended spectrum β-lactamase gene *bla*_CTX-M-15_, 61.1% (*n* = 33) harbored the non-Extended Spectrum Beta-Lactamase(ESBL) gene variant *bla*_TEM-1_, while in another three isolates the *bla*_TEM_ variants could not be resolved. A total of 50% (*n* = 27) were also found to harbor the *bla*_SHV_ gene. Sequencing revealed the presence of two ESBL *bla*_SHV_ gene variants (*bla*_SHV12_ (*n* = 1; 3.7%) and *bla*_SHV27_ (*n* = 8; 29.6%)), along with six non-ESBL variants (*bla*_SHV1_ (*n* = 8; 29.6%), *bla*_SHV33_ (*n* = 3; 11.1%), *bla*_SHV85_ (*n* = 3; 11.1%), *bla*_SHV108_ (*n* = 1; 3.7%), *bla*_SHV123_ (*n* = 1; 3.7%), and *bla*_SHV180_ (*n* = 1; 3.7%)). One *bla*_SHV_ variant could not be resolved. Among the nine ESBL *bla*_SHV_ gene variants, four each were found in *A. baumannii* and *K. pneumoniae* isolates and one in an *E. coli* isolate. However, there were no differences in the distributions of resistance phenotypes against cefotaxime and P/T among the three species, and a comparison of β-lactamase gene carriage profiles revealed significantly increased occurrences of the *bla*_CTX-M-15_ gene among the *A. baumannii* isolates and decreased *bla*_SHV_ gene occurrences among the *E. coli* isolates (*n* = 4; 20%) (Figure 2B, Table 4). None of the colistin-resistant HAA isolates were found to harbor the *mcr-1* gene (Table S1).

### 3.4. Distribution of the bla_NDM_ Gene and MBL Production among All the Isolates

The *bla*_NDM_ gene was not detected in all the meropenem non-susceptible isolates. A total of 55.7% (*n* = 39) HAA and 46.2% (*n* = 6) EWS isolates were found to be producing MBL, and a fraction of 68.9% (*n* = 31) harbored the *bla*_NDM_ gene. The *bla*_NDM_ gene was detected in a total of 19 (79.2%) meropenem resistant isolates and 13 (76.5%) intermediate isolates. However, there were 15 meropenem sensitive isolates that were found to be *bla*_NDM_ positive, of which 46.7% (*n* = 7) were identified to be *A. baumannii*. The *bla*_NDM_ gene subtypes identified were *bla*_NDM-1_ (*n* = 19; 45.2%), *bla*_NDM-2_ (*n* = 6; 14.3%), and *bla*_NDM-5_ (*n* = 17; 40.5%). Subtyping could not be carried out for six isolates, which had low band intensities. A species-specific clustering of *bla*_NDM_ gene variants was also observed. The *K. pneumoniae* isolates harbored more *bla*_NDM-1_ variants (80%) in comparison with the *E. coli* isolates, which predominantly harbored the *bla*_NDM-5_ variant (90.9%). 

### 3.5. Colistin Resistance Among the Carbapenem-Hydrolyzing HAA Isolates 

Given that carbapenem non-susceptible bacterial infections are often treated with colistin, the distributions of colistin resistance among meropenem susceptible and non-susceptible HAA isolates were compared. When both carbapenem-hydrolyzing, as well as the susceptible HAA isolates, harboring the *bla*_NDM_ gene were taken together (*n* = 56), a significantly higher (83.9%; *p*-value = 0.0033, Fisher’s exact test) number of colistin resistant isolates were observed in comparison with those isolates which neither exhibited meropenem non-susceptibility nor harbored the *bla*_NDM_ gene (*n* = 14; 42.9%). 

## 4. Discussion

Across the globe, infections associated with contaminated drinking water kills approximately 1800 children under the age of five every day [32]. It has also been reported by a number of countries that pathogenic microorganisms released in hospital wastewater contribute majorly to this contamination [33,34]. The WHO recommendation for hospital wastewater treatment entails three rounds of treatments, ending in disinfection of pathogens [35]. In spite of that, reports on the presence of MDR pathogens in hospital wastewater has been published by a number of countries [14,36,37], and there are a few reports from India as well [19,20]. Further, persistence of antibiotic-resistant bacteria in treated sewage water is also increasingly being reported because of the increase in pathogen loads within expanding communities and cities [38]. Thus, it has become imperative to understand and monitor antibiotic concentrations and pathogen loads in hospital sewage for appropriate water treatment before it is released to the environment or used for irrigation. 

The present study was undertaken in one of the most populated states of India [39] and the findings highlight the presence of WHO priority pathogens, namely, carbapenem non-susceptible *A. baumannii* and carbapenem non-susceptible, ESBL-producing *E. coli* and *K. pneumoniae*. Mirroring these findings, earlier reports on patient blood cultures collected from all over India have also shown high levels of resistance to aminoglycosides and third generation cephalosporins among *A. baumannii*, *E. coli*, and *K. pneumoniae* isolates [40]. However, in this study, it was observed that amikacin and meropenem non-susceptibility were the highest among *E. coli* isolates, which was in contrast to a 2018 report from New Delhi that had highlighted the presence of higher numbers of carbapenem-resistance in *A. baumannii* and *K. pneumoniae* isolates collected from small and large hospital wastewater out-falls, respectively [19]. 

In recent years, to tackle the rapid increase in prevalence of carbapenem-resistant isolates in human infections, the use of the relatively toxic polymyxins has become more frequent, and multiple studies from various parts of the world have also confirmed the parallel increase in dissemination of colistin-resistant isolates in wastewater and water bodies [41,42,43,44,45]. Regardless of an increase in polymyxin sales in India between 2000–2015 [4], data on dissemination has been lacking. The high prevalence of colistin resistance (85.37%) observed in this study among the meropenem non-susceptible HAA isolates abundantly resonates the after effects of such usage practices. However, the absence of the *mcr-1* gene among the colistin-resistant isolates warrants further investigation to identify the resistance determinants. 

The presence of the *bla*_NDM_ gene in 78.05% of the meropenem non-susceptible isolates in the present study is indicative of the presence of other carbapenemase genes. Additionally, the observed *bla*_NDM_ variant types and frequencies were also distinct from the previous studies [16,19]. Interestingly, a total of 21.4% of the meropenem-sensitive isolates were both MBL-producers and harbored the *bla*_NDM_ gene, which requires further analyses of gene copy number variations among the isolates. The CTX-M-15 variant, prevalently found in the Enterobactericeae family and known to be a dominant variant in India, was also found to be the predominant ESBL among the HAA *A. baumannii*, *K. pneumoniae*, and *E. coli* isolates in this study [46]. Further, it was interesting to note that all the *E. coli* isolates harboring the *bla*_CTX-M-15_ gene were also non-susceptible to ciprofloxacin, a second generation fluoroquinone, which is a known characteristic of the sequence type (ST) 131 clone [47]. However, a 2017 report using whole genome sequences of extraintestinal pathogenic isolates have indicated the spread of the CTX-M-15 gene variant to other STs as well [48]. Hence, further analyses for accurate clonal characterization will be necessary to identify the ST clones included in this study. Similarly, high CTX-M-15 carriage rates among the *A. baumannii* isolates, as observed in this study, have been reported earlier among clinical *A. baumannii* isolates [49], although the rise in dissemination might be attributed to the excessive use of third generation cephalosporins. 

## 5. Conclusions

Taken together, this study, in spite of a modest sample size and single season collection, provides evidence for the presence of large proportions of colistin-resistant and carbapenem-hydrolyzing human pathogens enlisted in the WHO priority list for the dearth of therapeutic options. Given the presence of overcrowding in the populated districts included in the study and warm climatic conditions, the spread of such pathogens and human exposure can have serious consequences. Wider implications of these findings are yet to be seen. However, the spread of nosocomial pathogens to the environment may also result in the transfer of antimicrobial resistance genes to environmental isolates, which can further worsen the present scenario. Hence, it is imperative that hospital wastewater be monitored and treated adequately by hospitals before being released to common treatment plants. 

## Figures and Tables

**Table 1:** Description of the water samples included in this study.

Location ID	Water Source	District (City/Town)	Date of Collection	pH
W.KL.JN1	Hospital wastewater outfall	Nadia (Kalyani)	2.5.2018	8.12
W.KL.GH1	Hospital wastewater outfall	Nadia (Kalyani)	2.5.2018	7.92
W.CGR.SD1	Hospital wastewater outfall	Hoogly (Chandannagar)	8.5.2018	8.44
W.CNS.ISH1	Hospital wastewater outfall	Hoogly (Chinsurah)	8.5.2018	7.88
W.HWH.DH1	Hospital wastewater outfall	Howrah	11.5.2018	7.53
W.B.ES1	Hospital wastewater outfall	Howrah (Belur)	11.5.2018	7.91
W.KOL.MC1	Hospital wastewater outfall	Kolkata	16.5.2018	7.93
W.KOL.NR1	Hospital wastewater outfall	Kolkata	16.5.2018	7.85
W.KOL.NRP1	Hospital Pond	Kolkata	16.5.2018	9.54
W.KH.SDP1	Hospital Pond	North 24 Parganas	16.5.2018	8.15
W.KH.ES1	Hospital wastewater outfall	North 24 Parganas	16.5.2018	7.44
W.KD.BH1	Hospital wastewater outfall	North 24 Parganas	16.5.2018	7.39
W.BNG.PW	Pond	North 24 Parganas (Bonhoogly)	21.5.2018	8.19
W.KL.L1	Lake	Nadia (Kalyani)	2.5.2018	7.87
W.CGR.P1	Pond	Hoogly (Chandannagar)	8.5.2018	8.53
W.B.P1	Pond	Howrah (Chandmari)	11.5.2018	8.56

**Table 2:** Differences in the antimicrobial non-susceptibilities between HAA (*n* = 70) and EWS (*n* = 13) bacterial isolates.

Antimicrobials	HAA Isolates (%)	EWS Isolates (%)	*p*-Value	False Discovery Rate (FDR) of 0.05
Cefotaxime	69 (98.6)	11 (84.6)	0.0625	0.0429
Piperacillin/Tazobactam	67 (95.7)	8 (61.5)	0.0019 *	0.0286
Meropenem	41 (58.6)	0 (0)	0.0001 *	0.0071
Ciprofloxacin	45 (64.3)	1 (7.7)	0.0002 *	0.0143
Gentamicin	19 (27.1)	0 (0)	0.0332 *	0.0357
Amikacin	45 (64.3)	2 (15.4)	0.0016 *	0.0214
Chloramphenicol	14 (20)	0 (0)	0.1117	0.0500

* Statistically significant *p*-values are highlighted in bold.

**Table 3:** Differences in the antimicrobial non-susceptibilities between HAA *A. baumannii* (*n* = 16), *E. coli* (*n* = 20), and *K. pneumoniae* (*n* = 18) isolates.

Antimicrobials	*A. baumannii* (%)	*E. coli* (%)	*K. pneumoniae* (%)	*p*-Value	FDR of 0.05
Cefotaxime	16 (100)	19 (95)	18 (100)	1.00000	0.0438
Piperacillin/Tazobactam	15 (93.8)	19 (95)	17 (94.4)	1.00000	0.0500
Meropenem	4 (25)	16 (80)	10 (55.6)	0.00432 *	0.0125
Ciprofloxacin	9 (56.3)	18 (90)	10 (55.6)	0.03337	0.0188
Gentamicin	2 (12.5)	9 (45)	4 (22.2)	0.07808	0.0313
Amikacin	4 (25)	18 (90)	11 (61.1)	0.00037 *	0.0063
Chloramphenicol	7 (43.8)	3 (15)	2 (11.1)	0.06227	0.0250
Colistin	12 (75)	14 (70)	15 (83.3)	0.66841	0.0375

* Statistically significant *p*-values are highlighted in bold.

**Table 4:** Differences in *β*-*lactamase* gene carriage.

β-Lactamase Genes	*A. baumannii* (%)	*E. coli* (%)	*K. pneumoniae* (%)	*p*-Value	FDR of 0.05
*bla*_CTX-M-15_	14 (87.5)	8 (40)	8 (44.4)	0.00878 *	0.0333
*bla*_TEM_	13 (81.3)	15 (75)	8 (44.4)	0.04596	0.0500
*bla*_SHV_	9 (56.3)	4 (20)	15 (83.3)	0.00045 *	0.0167

* Statistically significant *p*-values are highlighted in bold.

## Supplementary Materials

The following are available online at https://www.mdpi.com/1660-4601/17/3/1007/s1, Table S1: Characteristics of all the isolates included in the study. Table S2: Differences in the distribution of amikacin and meropenem non-susceptibility among HAA *A. baumannii* (*n* = 16), *E. coli* (*n* = 20), and *K. pneumoniae* (*n* = 18) isolates (Post-hoc tests).
